# Laser-Assisted Management of a Rare Presentation of Peripheral Ossifying Fibroma in an Infant

**DOI:** 10.7759/cureus.20417

**Published:** 2021-12-14

**Authors:** Keerthi K Nair, Esha Nausheen, Kanad Chaudhuri, Madhu Hariharan, Sawen Ramesh

**Affiliations:** 1 Department of Dentistry, Dr. Madhu's Centre for Laser Dentistry and Endodontics, Kochi, IND; 2 Department of Dentistry, Murshidabad Medical College & Hospital, Berhampore, IND

**Keywords:** photobiomodulation, epulis, natal tooth, laser-assisted excision, reactive lesions in infants

## Abstract

Reactive hyperplasias are a group of lesions often seen in the oral mucosa, especially on the gingiva, in association with local irritation or trauma. Peripheral ossifying fibroma (POF) is a common reactive lesion, almost always affecting the tooth-bearing areas of the oral cavity. It is most often encountered in young adults but extremely rare in patients below 10 years of age.^ ^Here, we report a unique presentation of peripheral ossifying fibroma affecting the anterior mandible in a three-month-old infant. We also highlight the role of laser in the management of such lesions.

## Introduction

The gingiva is susceptible to a wide range of irritants, and tissue response to these irritants may vary [1]. According to Buchner et al. (2010), localized hyperplastic reactive lesions of the gingiva include focal fibrous hyperplasia (FFH), pyogenic granuloma (PG), peripheral giant cell granuloma (PGCG), and peripheral ossifying fibroma (POF) [2]. POF presents itself as a gingival epulis secondary to trauma or irritation [1]. Its occurrence in infants is exceedingly rare and hence diagnosis and management can be challenging for the clinician. The present case was managed by laser-assisted excision and photobiomodulation; here, we emphasize the benefits of the same.

## Case presentation

A three-month-old female infant was referred to our dental unit for the management of a lump in her lower front gum pad, with associated difficulty in feeding. According to her parents, she had two teeth in the lower anterior region of the mouth at birth. As the teeth were superficially present, they were removed on the same day using gauze and tweezers. Postoperative bleeding was minimal. A few days later, they observed a small lump at the same site, which slowly increased to the present size. It was initially pale pink, which turned into a reddish-purple following a minor trauma three days earlier. The baby later became very irritable and refused direct breastfeeding. The baby was otherwise healthy and was born following a full-term pregnancy without any complications. There was no history of consanguinity, and antenatal history was unremarkable. The parents first consulted a pediatrician regarding the problem, who then referred her to our dental unit for diagnosis and management.

**Figure 1:**
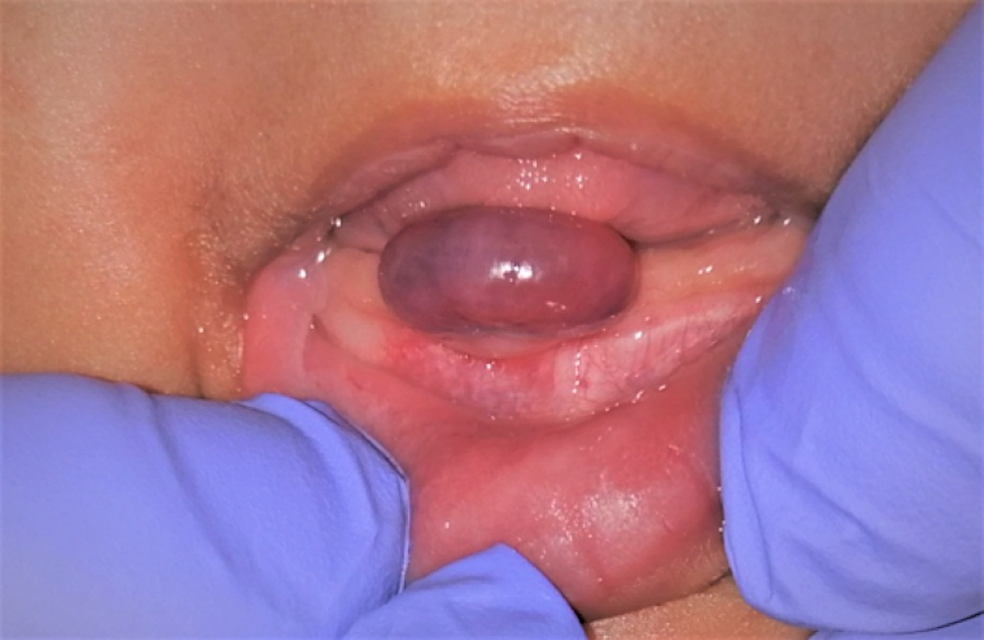
Intraoral photograph revealing a reddish-purple nodular swelling, measuring approximately 0.5 × 1 cm with the shape of a kidney bean in the anterior mandibular ridge

The extraoral examination did not reveal anything of clinical significance. Intraoral examination revealed solitary pedunculated nodular swelling in the mandibular anterior gum pad. The swelling appeared reddish-purple, measuring approximately 0.5 × 1 cm in size, and had the shape of a kidney bean. The mucosa over the swelling appeared tense and shiny. The surrounding mucosa seemed to be healthy. The swelling was tender, soft to firm in consistency, and mobile. The swelling was non-fluctuant and nonpulsatile and was not readily bleeding on manipulation, and the blanch test was negative (Figure 1). A provisional diagnosis of epulis involving mandibular anterior gum pad was made, and irritation fibroma, pyogenic granuloma, and congenital epulis were listed as the differential diagnoses.

Routine blood investigation reports were within the normal limits for the age and sex of the patient. A laser-assisted excision was planned under local anesthesia after obtaining informed consent from the parents. A diode laser of 940 nm wavelength (λ) in 1 W power was used for the complete excision of the lesion, following necessary safety protocols.

**Figure 2:**
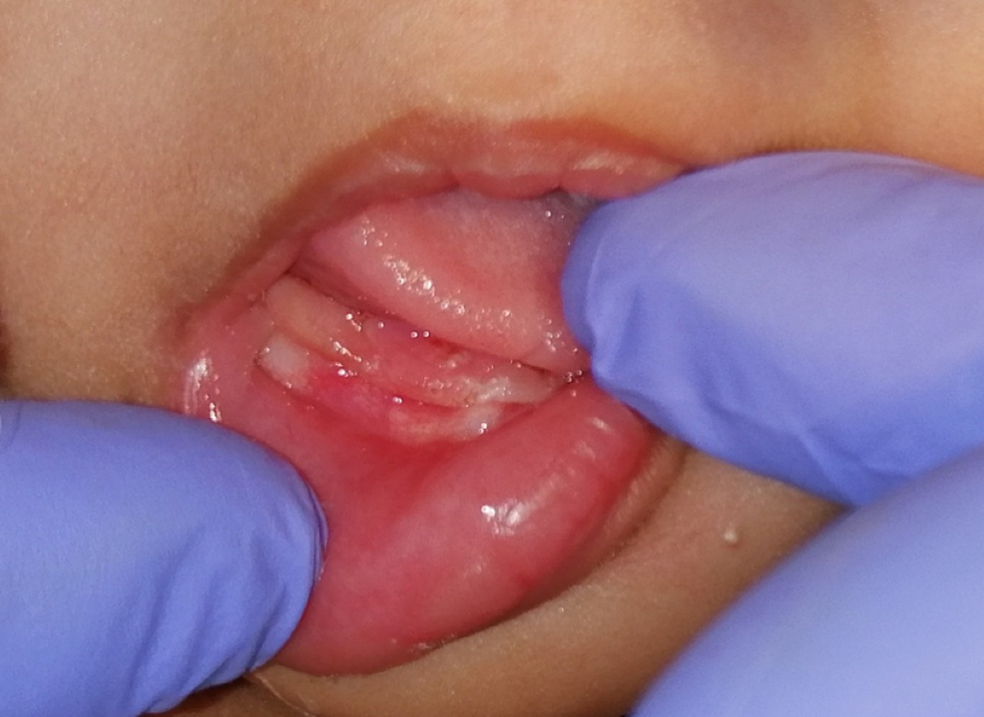
Immediate postoperative photograph demonstrating complete excision and excellent hemostasis

**Figure 3:**
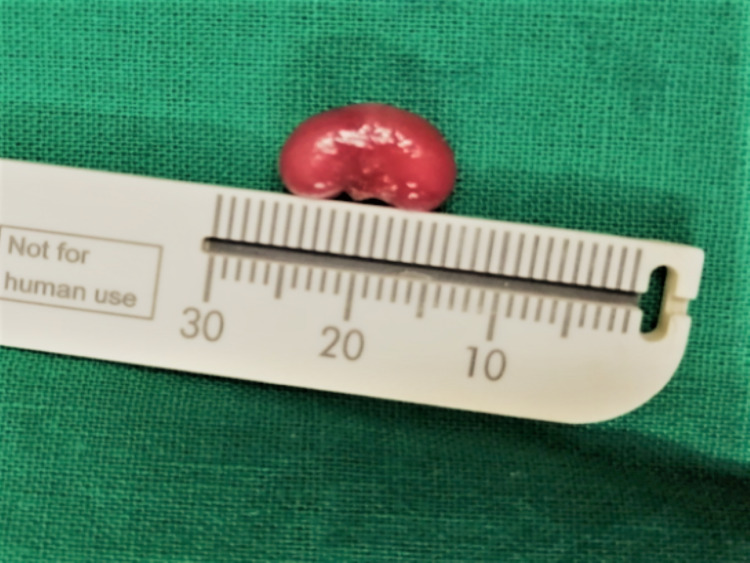
Gross specimen appeared in the shape of a kidney bean, measuring 1 × 0.5 cm

The procedure was uneventful with excellent hemostasis (Figure 2), and the gross specimen (Figure 3) was sent for histopathological assessment. The surgical site was subjected to photobiomodulation in the immediate postoperative period in an attempt to fasten healing and reduce postoperative complications. Diode laser with 660 nm wavelength (λ) in 360 mV power was used for one minute, with a total energy of around 2 J delivered at the surgical site. No postsurgical medications were given. The patient was reviewed after three days. She was asymptomatic and was taking direct breastfeeding, and the wound was healing well.

**Figure 4:**
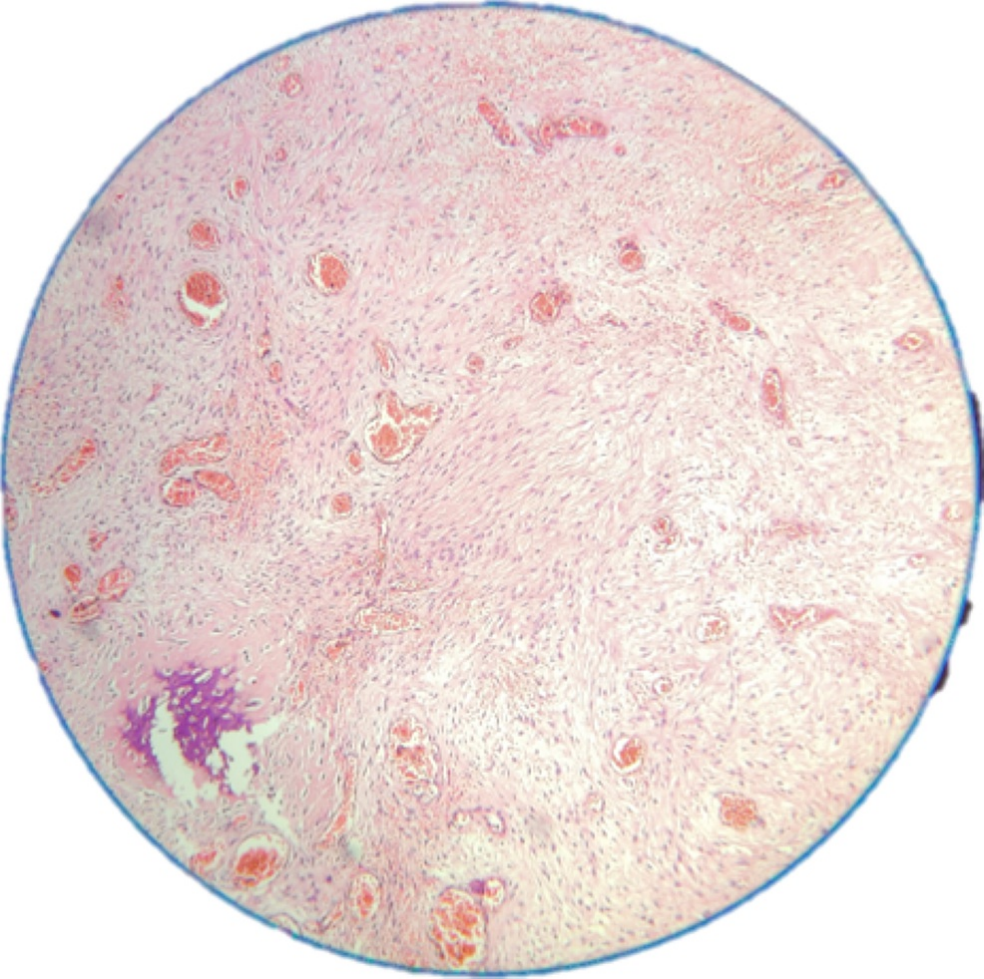
Photomicrograph revealing spindle to stellate cells with hyperchromatic nuclei in a loose myxoid and fibrous background with no evidence of mitosis (10×)

**Figure 5:**
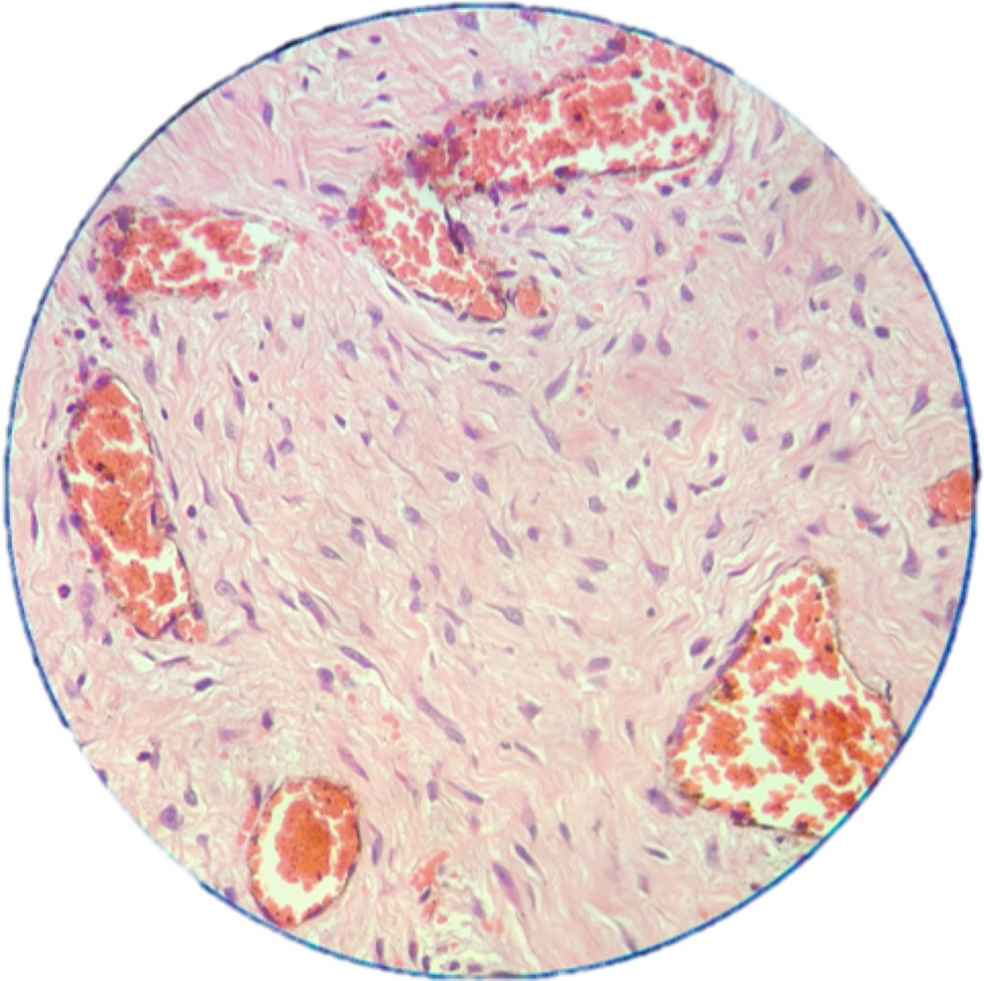
Photomicrograph showing evidence of congested blood vessels and few bony trabeculae (40×)

Histopathological study revealed spindle to stellate cells with hyperchromatic nuclei in a loose myxoid and fibrous background with no evidence of mitosis (Figure 4). There was evidence of numerous congested vessels and a few bony trabeculae (Figure 5).

**Figure 6:**
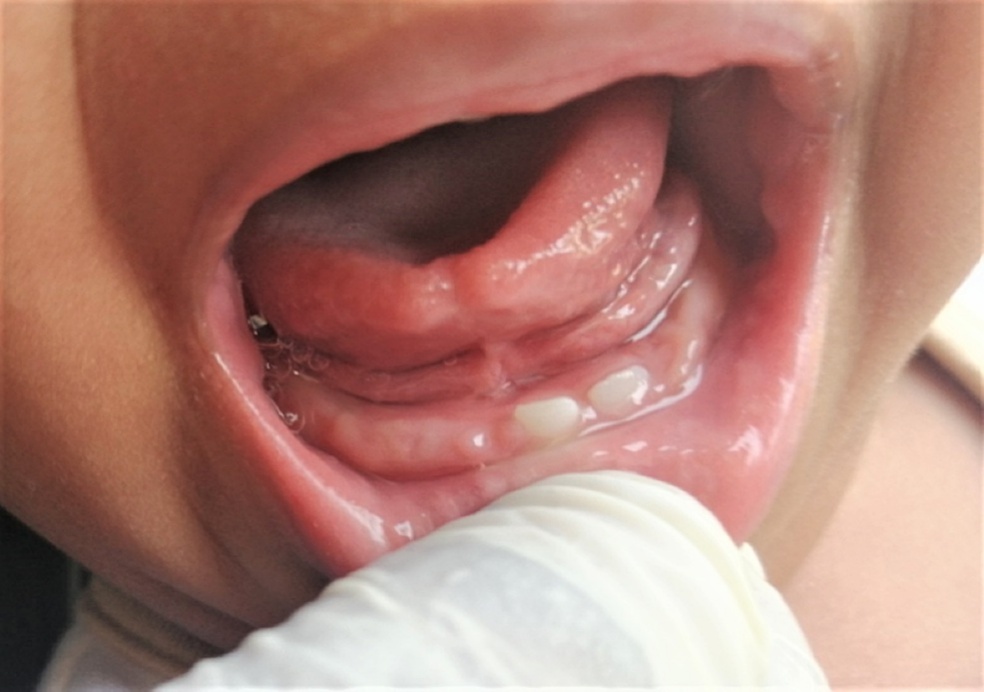
Photograph showing no evidence of recurrence at sixth month (postoperative) follow-up and presence of deciduous teeth w.r.t 71 and 72

A final diagnosis of peripheral ossifying fibroma of mandibular anterior gum pad was made. On follow-up, a week later, complete healing of the surgical site was observed. There was no recurrence in six months of follow-up (Figure 6).

## Discussion

POF is a distinct clinical entity characterized as focal, reactive, nonneoplastic, tumorlike growth arising from the gingiva [1]. The pathogenesis of POF is uncertain. It is now considered as a unique entity originating from the undifferentiated mesenchymal cells of the periodontal ligament or periosteum rather than a transitional form of pyogenic granuloma or irritation fibroma [3,4]. Local irritation or trauma is speculated to trigger the hyperreactivity of progenitor cells of the periodontal ligament [4-6], and certain endocrine hormone levels in the serum are thought to influence this tissue response [5]. Immunohistochemical studies have further validated the local irritation theory [7]. The lesion can occur in edentulous areas and is proposed to arise due to chronic irritation from the affected tooth before extraction or triggered by extraction [8].

POF is found to be common in females and often encountered during pregnancy [9]. The influence of hormonal factors in the etiopathogenesis of POF has been considered responsible for the higher prevalence in females [10]. A peak of incidence of POF is seen in the second and third decades of life and is rare in very young and older adults. The prevalence rate in the 0-10 years age group has been reported to be as low as 1%-2% [1,2,9].

POF usually presents as a solitary, pedunculated or sessile, slow-growing, painless, nodular mass, often less than 2 cm in size, mostly located in the interdental papilla [5]. POF is found most often in the anterior maxilla, the color of the lesion can be pink or at times erythematous, and the surface may appear smooth or ulcerated [11]. Pain, ulceration, displacement of the adjacent teeth, and resorption of the underlying alveolar bone have been reported to be associated with large or multicentric lesions. Radiographically varying amounts of erosion can be seen in the alveolar bone depending on the size of the lesion, maybe with some amount of mineralization in the lesional matrix [7]. A final diagnosis of POF is drawn based on histopathological analysis [11].

The tooth that is present since birth is a natal tooth, whereas the one that erupts in the first month of life is termed a neonatal tooth. Local reactive lesions in infants are infrequent and are mostly in association with a natal or a neonatal tooth [7]. It is postulated that active growth in the infant jaw gets trigged by the trauma caused by the removal of the natal tooth, leading to an exuberant periosteal response, resulting in a reactive lesion with some production of bone [9]. POF is a reactive lesion with a sporadic occurrence in infants, and to the best of our knowledge, only five cases have been reported previously (Table 1) [7,12-15].

**Table 1:** Review of cases reported in the literature CP: clinical presentation

Reported cases	Age/gender	Clinical features	History of natal/neonatal tooth	Differential diagnosis	Management, follow-up, and recurrence
Tewari et al. (2016) [7]	Two months/M	Site: anterior mandibular ridge; size: 2.5 × 1 × 1 cm; CP: pink, nodular, pedunculated mass with smooth intact surface and nontender	Yes (neonatal tooth)	–	940 nm diode laser-assisted excision; 18 months, no recurrence
Yip et al. (1973) [12]	Seven days/F	Site: maxillary right posterior ridge; size: 1.5 × 1 cm; CP: soft pedunculated swelling	No	–	Surgical excision; on five months follow-up, no recurrence
Kohli et al. (1998) [13]	Two hours/F	Site: anterior mandibular ridge; size: 2 × 1.2 × 0.6 cm; CP: soft fluid pink fluctuant mass since birth	Yes (neonatal tooth at two weeks)	–	Surgical excision at the age of four weeks; two weeks, no recurrence
Acharya et al. (2015) [14]	Three months/F	Site: anterior mandibular ridge; size: 0.5 × 1.5 cm; CP: sessile, nodular, reddish-pink mass with white areas, tender and rubbery in consistency	Yes (natal tooth)	Irritation fibroma, pyogenic granuloma, peripheral giant cell granuloma	Surgical excision under local anesthesia; three months, no recurrence
Schafer et al. (2019)[15]	3.5 months/M	Site: anterior mandibular ridge; size: 7 × 6 mm; CP: round, pedunculated papule, slightly fluctuant, non-ulcerated, and with slightly purple discoloration	Yes (natal tooth)	Congenital epulis, eruption hamartoma, fibroma	Surgical excision; 12 months, no recurrence

Similar to the present case, all the cases reported previously were associated with natal or neonatal teeth and presented mostly in the anterior mandibular ridge. However, in a case reported by Yip et al. (1973), there was no association with either natal or neonatal tooth, and the site was at the posterior maxilla [12].

In adults, POF needs to be differentiated from other reactive lesions such as PG, PGCG, traumatic fibroma, and peripheral odontogenic fibroma [2]. However, when POF presents as a lesion in the alveolar ridge in infants, the differential diagnosis varies. Soft tissue tumors such as congenital epulis, vascular malformations such as hemangioma, or lymphangioma are considered. Congenital epulis is an extremely rare condition and can present at or immediately after birth. Prenatal 3D imaging such as ultrasound and MRI can give diagnostic clues by the 36th week of gestation. Congenital epulis has a striking predominance for females and appears as pink, smooth to lobulated, pedunculated mass. Unlike POF, it occurs mostly in the maxillary canine alveolar ridge region and is not often associated with a natal tooth [16]. Although intraoral lymphangiomas are rare, the common location is the dorsal surface of the tongue. Intraoral lymphangiomas present as distinct vesicles and may be superficial, which will have a granular or translucent appearance. Damage of overlying blood capillaries may result in the bluish-red appearance of the lesion [17]. Hemangiomas classically manifest as reddish-blue discoloration and may show hyperthermic changes. Unlike hemangioma, lymphangioma seldom undergoes spontaneous regression [17].

Complete surgical excision and follow-up are a necessity due to the known recurrence of POF. An alternative to conventional scalpel excision, lasers such as the CO_2_, neodymium-doped yttrium aluminum garnet (Nd:YAG), and diode have been used in mucogingival surgeries and excision of intraoral soft tissue lesions as an advanced and effective treatment modality [10,18].

Minor surgical procedures for infants are often traumatic for the child as well as parents and might require intervention under general anesthesia. In 2013, the American Academy of Pediatric Dentistry recommended lasers as an alternative and complementary method for providing soft and hard tissue dental procedures in infants, children, adolescents, and persons with special healthcare needs [19]. Excision of soft tissue lesions with laser offers better precision, hemostasis, clear operating field, minimal anesthesia, no requirement of sutures, superior patient acceptance, lesser procedure time, reduced healing time, and lower postoperative discomfort [10,18,19]. The postoperative requirements of analgesics are usually minimal, and the bactericidal effects of lasers on tissue minimize the need for antibiotics [20].

In the present case, we chose a diode laser with a 940 nm wavelength, considering its precision, safety, and deeper penetration for complete excision. Hemoglobin is one of the target chromophores for diode lasers; hence, they provide superior hemostasis [10,18].

A literature review shows that low-level laser therapy (LLLT) is an effective, noninvasive, and safe adjuvant aid in reducing postoperative complications, providing satisfactory analgesia, and also fastening the healing process [20]. When irradiated to the surgical site, a low-level laser causes photobiomodulation of the tissue operated, leading to the resolution of the inflammatory process with tissue repair. Studies have demonstrated the effectiveness of photobiomodulation in the immediate postoperative period even in neonates [20]. We chose a 660 nm laser in the immediate postoperative phase for bringing its photobiomodulation effect on operated tissues.

## Conclusions

Infants presenting with an intraoral swelling require immediate attention as it can affect nutrition and quality of life directly. The present case suggests that a thorough understanding of common, as well as rare, pediatric oral lesions are mandatory for drawing possible differential diagnoses, thereby providing prudent management. Parents’ anxiety and apprehension often demand counseling and reassurance. Laser-assisted excision provides advanced, effective, and easy management of such lesions with minimal operative and postoperative discomfort. In addition, photobiomodulation in the immediate postoperative phase has got promising results in further preventing postoperative complications and aiding in tissue repair. Regular follow-up of the patient is necessary due to known recurrence of POF, although it is rare after complete excision.
